# Black oil sunflower seed ingestion and suspected acute lipid toxicity in 4 alpacas

**DOI:** 10.1016/j.vas.2025.100438

**Published:** 2025-03-03

**Authors:** Chelsea C. Pulter, Omar A. Gonzales-Viera, Beckie Perell, Emma Deane, Asli Mete

**Affiliations:** a California Animal Health and Food Safety Laboratory, UC Davis, 620 W Health Sciences Dr, Davis, CA 95616, USA; b Loomis Basin Equine Medical Center, 2973 Penryn Rd, Penryn, CA 95663, USA; c Loomis Alpacas, 3600 Holly Hill Ln, Loomis, CA 95650, USA

**Keywords:** Alpacas, South American Camelids, Black oil sunflower seeds, Fat toxicity, Lipid toxicity, Gastric ulcers, C1 ulcerations

## Abstract

Four adult female alpacas from the same property in Loomis, CA developed clinical signs of recumbency, lethargy, anorexia, and had abdominal pain at least 48 h after incidental ingestion of a large volume of black oil sunflower seeds. One alpaca died, one was euthanized and necropsied, and two alpacas were treated by Loomis Equine Medical Center. The necropsied alpaca was found to have ingested numerous black oil sunflower seeds along with erosion and ulceration of the distal esophagus, C1, and C2 chambers. Ancillary tests performed were without significant findings. Treatment for the suspected acute toxicity in two alpacas included IV fluids, injectable antibiotics, and activated charcoal by orogastric tube. Sunflower seeds and lipid containing fluid were recovered from one of the alpacas that was euthanized due to poor prognosis. Overall, three of the four alpacas died or were euthanized, and one survived with outpatient treatment.

Our current case series shows significant morbidity and high mortality from the ingestion of highly available lipid, in the form of easily shelled black oil sunflower seeds. The large amount of lipid inhibits the activity of the forestomach bacteria and coats the fibrous feed, overwhelming the body's ability to metabolize unsaturated lipids. We also surmise that the seed hulls are traumatic to the camelid's esophagus and forestomach mucosa causing mucosal ulceration and eventual sepsis. We conclude that excessive levels of lipid may be fatal in alpacas, and that diets with high lipid content such as black oil sunflower seeds should not be fed to camelids.

## Highlights

• A toxic amount of ingested lipid causes an acute syndrome and high mortality rate in South American camelids.
• South American camelids that ingest a high concentration of lipids all show the same clinical presentation and pathological signs.
• Insulin and activated charcoal may be beneficial in South American camelid lipid toxicity.

## 1 Introduction

This case series aims to increase awareness of camelid susceptibility to acute ingestion of high-fat seeds in diets, whether it is by accidental ingestion or added to the feed rations. We propose that an acute large ingestion of fats (as an oil) causes lipid toxicity and death in South American camelids (SACs). In these cases, the alpacas ingested unsecured black oil sunflower seeds (ad libitum, unknown volume) which were used to feed wild birds and as a supplement to chickens.

Black oil sunflower seeds (*Helianthus annuus*) are composed of unsaturated fatty acids (UFA) – mono oleic acid at 19.81 % and poly linoleic acid at 64.35 % (Petraru A et al., 2021). Most brands of black oil sunflower seeds contain 25 % - 38% min crude fat, in which the oil content is 50 % of the seed's dry weight. The seeds that were ingested were a commercially produced product with a minimum of 30 % crude fat content. Ruminants and extrapolated to “pseudo ruminants” (SACs) require only about 4–6 % of fat supplementation in their diet (Bionaz M et al., 2020).

## 2 Materials and methods

In May of 2023, 4 alpacas in a herd of 12 mature adults located in Loomis, CA, were found to be recumbent or cushed, lethargic and anorexic. The herd consisted of 1 male that is separated and 11 females, residing in a 1-acre irrigated Bermuda grass pasture with an enclosed barn. The alpacas were fed orchard or alfalfa hay freely, Bar Ale Alpaca concentrate pellets (18 % protein, 2.9 % Fat, 18.6 % fiber) and loose minerals daily. The black oil sunflower seeds (Bar Ale) were kept on the property as a supplement to chicken feed. The bag did not fit in the metal trashcan where it normally was kept, and it was left out with the top rolled down unsecured next to the can. The owner had left for a few days for vacation, and it was later noted that at least 4 alpacas had gained access to the seeds. It was unknown when the alpacas ingested the seeds, however, it was at least 2 days before the clinical signs were noted after the owner had returned.

Alpaca 1, a 55 kilogram 15-year-old female alpaca, was the first to show signs of illness and died shortly after. She had separated from the herd and did not eat any hay that was offered. The primary veterinarian suggested starting the alpaca on a digestive paste probiotic but later that night this alpaca was found cushed, and her breathing was tachypneic and shallow. Alpaca 1 died the following morning and was rendered.

Alpaca 2, a 59 kilogram 17-year-old female alpaca, was found to be cushed, and had very shallow and fast breathing early that morning. It was noted she was moaning, did not get up to eat, drink, or urinate and her abdomen was subjectively swollen. Alpaca 2 was euthanized in the field and submitted to the California Animal Health and Food Safety Laboratory System, Davis branch, UC Davis, for full necropsy and ancillary testing. No treatment was given to this alpaca. On necropsy, it was found that this animal ingested an innumerable amount of diffusely black-shelled sunflower seeds. Upon investigation by the owner, it was found that the seeds were black oil sunflower seeds she kept on the property for her chickens.

Alpaca 3, a 55 kilogram 14-year-old female alpaca, was also noted to be cushed, lethargic, anorexic and isolating herself from the herd. This alpaca was taken to a primary care practice, Loomis Basin Equine Medical Center to be treated later that morning. Her temperature was 93.5° Fahrenheit (99.5 – 100.5 Fahrenheit), heart rate was 112 beats per minute (60–90 beats per minute), respiration was 12 breaths per minute (10–30 breaths per minute), capillary refill time was elevated at 5 s (< 2 s) and mucous membranes were pink. Biochemistry was performed on plasma (from a heparinized sample) on in house analyzers (Heska Element HT5 and Element DC5X) and showed marked hyperlactatemia 7.65 mmol/L (0–1.4mmol/L), dehydration (PCV 45 % (22–40 %), total protein 7.4 g/dL (5.3–7.9 g/dL)), low bicarbonate 13.4 mmol/L (22–29 mmol/L) interpreted as metabolic acidosis (pH 7.359), hyponatremia 126 mEq/L, hypochloremia 82 mEq/L (102–120 mEq/L), hyperkalemia 5.7 mEq/L (2.4–4.6 mEq/L), hyperglycemia > 600 mg/dL(80–120 mg/dL), hyperalbuminemia 4.2 g/dl (2.8–3.9 g/dl), severe azotemia (BUN 110.1mg/dL (10–25 mg/dL), creatinine 7.0 mg/dL (0.7–1.8 mg/dL)), elevated aspartate aminotransferase 437 U/l (100–360U/l) , and elevated creatine kinase 465 IU/L (50–400IU/L).Other testing that was done for this alpaca included an electrocardiogram that showed sinus tachycardia (heart rate 108 beats per minute) and on brief abdominal/thoracic ultrasound, the most notable finding was significantly edematous C1 compartment visualized on the left ventral abdomen (identified by the presence of saccules) measuring ∼9–10 mms (normal GI wall thickness is ∼3 mm) on the ventral floor of the abdomen. The thorax was largely unremarkable. Alpaca 3 was hospitalized for suspected toxicity, severe acute kidney injury, and severe dehydration. An IV catheter was placed in the right jugular vein, a 3 L fluid bolus (Plasmalyte 148) (40ml/kg), and Ceftiofur sodium antibiotic (2.2mg/kg) were given intravenously and then placed on an isotonic bicarbonate drip at 150ml/hour thereafter (3ml/hr). Alpaca 3 was sedated with 10 mg of Midazolam HCl and a large bore orogastric tube was passed. A net volume of 9 L of foul-smelling, pearlescent reflux was removed via gastric lavage with several seeds that did not cause obstruction of the tube. Two cups of sunflower seeds were within the reflux fluid. 56.7 grams of Nutricost Activated charcoal mixed with 500 mL of water was administered via the orogastric tube and the tube was removed. The decision was made multiple hours later to euthanize due to poor response to treatment, poor prognosis and financial reasons.

Alpaca 4, a 60 kilogram18-year-old female alpaca was taken to the Loomis Basin Equine Medical Center in the afternoon on the same day, due to lethargy, abdominal pain, and anorexia. This alpaca was also treated symptomatically given similar clinical signs to the other alpacas. Alpaca 4′s physical examination showed tachycardia (88 beats per minute) and tachypnea (40 breaths per minute), normal rectal temperature, and absent gut sounds. Biochemistry was performed on plasma (from a heparinized sample) and showed hyperlactatemia, hypocalcemia, hyperglycemia, mild azotemia, and hematology showed leukocytosis (values unavailable). An orogastric tube was passed, and no net reflux was obtained. 60 grams (1g/kg) of activated charcoal with 500 mL water was given via orogastric tube and a single dose of Ceftiofur crystalline free acid (2.2mg/kg) was subcutaneously administered. It was recommended to keep the alpaca in the hospital on IV fluids due to suspected dehydration. The client elected to take the alpaca home to be treated. At home, the alpaca was given two additional doses of activated charcoal (60g ) and a digestive paste containing B vitamins and bacteria the next day. 6 days after clinical signs were first noted this alpaca started eating again. The client reported alpaca 4 had subjectively lost a significant amount of weight (values unavailable) but fully recovered approximately 3 weeks after the seed ingestion.

## 3 Results

### 3.1 Necropsy

**Fig. 1:**
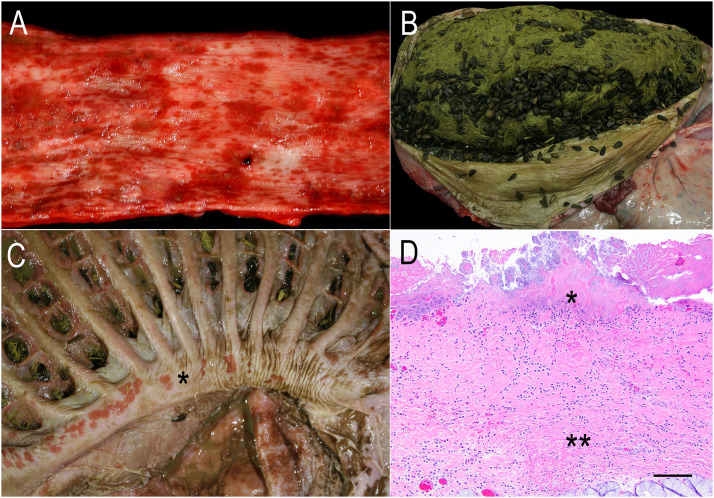
Multifocal to coalescing esophageal ulceration (A), abundant ingesta of hay and numerous black sunflower seeds (B), multifocal to coalescing ulcers in the pillar of C1 (asterisk) (C), and photomicrograph of a section of esophagus with superficial erosions and ulcers (asterisk) with necrotic debris and bacteria, as well as expansion of the submucosa by cellular infiltrate and fibrin (double asterisk) (D), H&E stain; bar = 100 µm in alpaca 2 with black oil sunflower seed over-ingestion.

Of the four alpacas with clinical signs, only one survived (Alpaca 4). It is likely that this animal had not ingested a high enough volume of sunflower seeds to cause significant disease and the supportive treatments for potential toxicity aided recovery. The other animal that was treated at the clinic but died (Alpaca 3), had a large volume of reflux of pearlescent fluid, indicative that digestion may have slowed down and contained a large volume of fats. There were 2 cups of sunflower hulls present within the fluid. Necropsy was performed on only one alpaca (Alpaca 2). The most significant necropsy findings were in the alpaca's esophagus and C1 compartment. The esophagus had severe ulceration of the distal half with empty (Fig. 1A), poorly masticated sunflower seed shells present at the gastroesophageal junction. Occupying the complete C1 and to a lesser extent C2 were a large number of diffusely black, thin-shelled, empty sunflower seed shells admixed with hay (Fig. 1B). Widespread coalescent ulcers were seen throughout the mucosa of C1 (Fig. 1C) and to a lesser extent in C2. The pH of C1 was 7.0. C3 had ulcers present in the distal part (a common necropsy finding in SACs, considered to be associated with stress). As general practice on necropsy, blood and tissues were tested for bacterial involvement by cultures of the lung and liver, for bovine viral diarrhea virus, border disease virus, and bluetongue virus by PCR, and the liver was screened for heavy metals and selenium concentration. All testing was completed by the CAHFS laboratory and there were no significant findings, including the isolation of *E. coli* in the lung tissue as a secondary finding (data not shown).

### 3.2 Histopathology

Histopathology was carried out on the gastrointestinal tissues as well as the heart, lung, liver, kidneys, adrenal glands, spleen, skin and brain. Histologically, the esophageal and C1 mucosae had large segments of ulceration, necrosis, frequently scattered fibrin thrombi, and bacterial colonization (Fig. 1D). The lungs had mild pulmonary edema and alveolar fibrin exudation, considered incidental. The kidneys had mild to moderate pale hyaline casts in cortical tubules and mineralization scattered within medullary renal tubules, the latter typically considered secondary to dehydration and often a background lesion when it is mild and scattered in renal medullary ducts. There were no microscopic changes in the remainder of the examined organs.

## 4 Discussion

### 4.1 Lipid toxicity theory

The clinical findings in all four alpacas exposed to ad libitum black oil sunflower seeds included lethargy, anorexia, decreased forestomach motility, and pain. In this case series, abundant black oil sunflower seed hulls were observed in the C1 and C2 of alpaca 2 and in smaller numbers within the large volume of pearlescent fluid in Alpaca 3 that indicates that a high level of fat was ingested. The necropsy findings of ulcerative gastric lesions in Alpaca 2 are similar to grain overload in ruminants but do not include the main finding of a low pH. This is more suggestive of a hyperacute mechanism of action, or, in this case, possibly a separate mechanism related to rapid high-volume lipid ingestion.

Sunflower seeds are composed primarily of poly- and mono-unsaturated FAs, linoleic and oleic acid respectively, with a small amount of SFAs. Black oil sunflower seeds are hybrids produced from the end of 1900′s, that have a solid black hull, meaty seed, and high oil content. The high oil composition is mid-oleic (55–75 %) or, mostly, high-oleic acid (>85 %). Due to the easy shelling of the seed, it is a favorite to feed wild birds as it attracts many different species (National Sunflower Association, 2021). This ease of shelling likely contributes to the rapid release of lipid into the compartments upon ingestion, while leaving behind the shell composed of cellulose and lignin.

Information and data in the literature is lacking regarding microflora of camelids, however, it is typically assumed that the C1 in camelids and the rumen in ruminants are similar in microflora, mechanisms of metabolism, and microbial population dynamics (Fowler ME et al., 2010; Maia MR et al., 2010; Millen DD et al., 2016; Vargas et al., 2020). UFA's are toxic to ruminant forestomach microflora, and therefore to the C1 and C2 in SACs. The most likely mechanisms of toxicity/inhibitory mechanism/bacteriostatic of UFAs to bacteria are overloading of the cellular metabolism of the lipid digesting microflora, disruption of the wall of bacteria by attaching to the lipid bilayer, and biofilm formation of the roughage with UFA lipid thereby decreasing fibrous availability for digestion. Once lipids enter the rumen the microorganisms promote UFA hydrolysis and biohydrogenation rapidly (Maia MR et al., 2010; Millen DD et al., 2016; Vargas JE, 2020). Bacterial populations such as *Butyrivibrio fibrisolvens* that biohydrogenate UFAs to form saturated fatty acids (SFAs) may become paralyzed by the sudden rapid ingestion of the lipid. Biohydrogenation is a detoxifying process since SFAs are typically less toxic than UFAs (Maia MR et al., 2010; Millen DD et al., 2016). In a previous case study of six SACs (four alpacas, and two llamas), who had incidentally ingested a large volume of black oil sunflower seeds in 2021 (McKenzie et al., 2021) had significant morbidity and mortality. They investigated possible causes including testing for heavy metals, oxalates, vitamin D, and mycotoxins. Gas chromatography-mass spectrometry and liquid chromatography-mass spectrometry were used on the seeds to test for other potential toxins with no toxin identified. They hypothesized that the massive lipid ingestion could have played a role in the pathogenesis, given the large volume of seeds within the compartments. The clinical presentation and the pathologic findings in both case series are remarkably similar, including the ulcerative gastritis, renal changes and neutral pH. The significance of the renal histologic lesions are unknown at this time due to the absence of clinical work-up of Alpaca 2. Some mineralization in collecting ducts is often considered secondary to dehydration by diagnostic pathologists in our laboratory. However, the mild to moderate, scattered medullary tubular hyaline casts and the mineral deposits characteristic of dystrophic mineralization could be indicative of clinical disease, or at least, initiation of, given that the two other alpacas as well as the prior cases demonstrated renal disease on blood work. We hypothesize that massive lipid exposure of the C1 is the mechanism of toxicosis (Clark C et al., 2001; Van Saun RJ, 2009). While we hypothesize that the lipid ingestion is the inciting cause of disease, both case series have ulcerations in the esophagus and forestomach compartments. We also theorize that the sunflower seed hulls may cause further damage and ulceration to an already compromised mucosal lining of the esophagus and stomach compartments. This in turn can lead to anorexia, pain, bacterial translocation, sepsis and eventual death.

### 4.2 Possible treatments for lipid related metabolic conditions

SACs are commonly fed grasses that consist of approximately up to 4 % fats. In ruminant species, it is not recommended to feed more than 4–6 % of the diet (Bionaz M et al., 2020). What makes SACs unique is their ability to metabolize low-quality roughage into energy (Fowler ME et al., 2010; Van Saun RJ, 2009). They have metabolic adaptations to digest these forages such as urea recycling and high urease activity in the foregut fermentation vat which increases ammonia and helps microbes generate microbial protein. This allows the microbes to ferment low nitrogen content forages the camelids use as energy. This predisposes them to energy-related metabolic conditions. They also have higher blood glucose concentrations, a reduced ability to secrete insulin, and reduced tissue sensitivity to insulin. Camelids can rapidly mobilize body lipid stores promoting lipolysis and fatty acid released for oxidation into energy when in a negative energy balance. Due to this mechanism, they would not manage high-starch diets very well (Fowler ME et al., 2010; Van Saun RJ, 2009). Cebra et al. (2004) studied the effects of insulin and epinephrine on biochemical parameters in camelids, finding that insulin administration may be an effective treatment for disorders of fat mobilization. The research notes that epinephrine-induced hyperglycemia, ketonemia, and increased non-esterified fatty acids are similar to camelids with natural disorders of fat metabolism. Giving insulin could have applications to help the alpacas digest and use the ingested lipids (Millen DD et al., 2016). Alpaca 4′s chemistry showed hyperglycemia and leukocytosis which the primary care team attributed to stress (Section 2.1). Given the previous study and the knowledge that a high-fat feed was ingested, these parameters may indicate a metabolic disorder of fat, in which insulin administration could be helpful. The alpacas were also treated with activated charcoal due to suspected toxicant ingestion and it may have aided the recovery of Alpaca 4. Activated charcoal is a recommended treatment for any suspected ingested toxicant in ruminants. A study in mice suggests that activated charcoal can bind to lipids in the GI tract and may be a useful therapeutic tool for alpacas that ingest excessive amounts of fats (Zhang X et al., 2022). With the seriousness of the acute disease process, we should also consider gastric lavage and potentially surgical intervention. Complete decompression and emptying of the C1 to remove the sunflower seeds and fluid content is recommended. Though surgery in camelids is not without risks, such as gastric content contamination, sepsis, ileus, pneumonia and repeat distension of C1. Fully weighing the risks vs benefits is needed (Newman K, Anderson D, 2009).

## 5 Conclusion

There are opportunities here to advance our understanding of lipid metabolism, lipid toxicity and treatment in camelids through future research in the area. We need to further understand why a subjectively large ingestion of lipids can cause such severe acute signs. This can help primary care veterinarians build appropriate treatment plans early in the disease course. Unfortunately, the mortality rate was similar to the previous case series at 75 %, and therefore black oil sunflower seeds should be kept out of reach and not fed to camelids due to the potential for accidental over-ingestion, with high morbidity and mortality.

## Funding

This research did not receive any specific grant from funding agencies in the public, commercial or not-for-profit sectors.

## Ethics statement

*‘The authors confirm that the ethical policies of the journal, as noted on the journal's author guidelines page, have been adhered to. No ethical approval was required as this is a review/short communications article with no original research data.’*

## CRediT authorship contribution statement

**Chelsea C. Pulter:** Writing – review & editing, Writing – original draft, Visualization, Investigation, Formal analysis, Data curation. **Omar A. Gonzales-Viera:** Writing – review & editing, Data curation. **Beckie Perell:** Writing – review & editing, Investigation. **Emma Deane:** Writing – review & editing, Data curation. **Asli Mete:** Writing – review & editing, Supervision, Formal analysis, Data curation, Conceptualization.

## Declaration of competing interest

The authors declare that they have no known competing financial interests or personal relationships that could have appeared to influence the work reported in this paper.
